# Next-generation detection in bovine respiratory and enteric diseases: metagenomic and amplicon sequencing insights into microbial diversity

**DOI:** 10.3389/fvets.2026.1788101

**Published:** 2026-04-07

**Authors:** Zain Ul Abedien, Ian J. Lean, Steven P. Djordjevic, Paul M. Hick, Mark E. Westman, Janina Mckay-Demeler, John Webster, Barbara P. Brito

**Affiliations:** 1 Australian Institute for Microbiology & Infection, University of Technology Sydney, Ultimo, NSW, Australia; 2 School of Life and Environmental Sciences, Faculty of Science, The University of Sydney, Camden, NSW, Australia; 3 Scibus, Camden, NSW, Australia; 4 New South Wales Department of Primary Industries and Regional Development, Elizabeth Macarthur Agricultural Institute, Menangle, NSW, Australia

**Keywords:** bovine respiratory disease, calf enteric disease, infectious diseases, livestock, metagenomics

## Abstract

Respiratory and enteric diseases are major contributors to morbidity, mortality, and economic loss in cattle production, with significant implications for animal welfare, particularly in calves. Traditional diagnostic approaches have laid the foundation for pathogen detection in cattle, providing essential tools for disease surveillance and control. However, their targeted nature limits the capacity to identify unexpected, novel, or polymicrobial infections that often underlie complex respiratory and enteric syndromes. Recent advances in molecular technologies, particularly amplicon sequencing (metataxonomics), metagenomics, and metatranscriptomics, enable untargeted, high-resolution profiling of microbial communities directly from clinical samples, offering transformative potential for research and diagnostics. This review synthesises current applications of these approaches in bovine respiratory and enteric disease research, highlighting key findings across virology, bacteriology, and parasitology. Collectively, these studies have expanded the catalogue of the microbial diversity, yet their interpretation remains challenged by the still-evolving understanding of microbial contributions to pathogenesis. Progress toward clinical integration is further hindered by the need for methodological standardisation, validation, and improved interpretive frameworks. Looking ahead, advancing these technologies will require harmonised protocols, integration of multi-omics datasets, and robust experimental and epidemiological studies to establish causal links between microbial signatures and disease outcomes. By bridging discovery and application, these approaches hold the potential to enhance diagnostic accuracy, strengthen surveillance, and support sustainable cattle production systems. As these technologies continue to evolve, they are likely to play an increasingly central role in bovine disease research and diagnostics.

## Introduction

Respiratory and enteric diseases are among the leading causes of morbidity and mortality in cattle, exerting a substantial toll on both beef and dairy industries (1). Young calves are particularly vulnerable, with gastrointestinal and respiratory infections contributing to significant production losses and animal welfare concerns (2, 3). Enteric diseases, often driven by viral, bacterial, or parasitic pathogens, are especially prevalent in neonates, with diarrhoea responsible for nearly half of all deaths in one-month-old dairy calves (4, 5). Similarly, bovine respiratory disease (BRD), a multifactorial syndrome marked by fever, anorexia, lethargy, coughing, and nasal or ocular discharge, can progress to pneumonia and death in animals of all ages. BRD is shaped by a complex interplay of environmental stressors and infectious agents, and its polymicrobial nature complicates both diagnosis and control across production systems (3).

Despite technological advances that have deepened our understanding of disease epidemiology and pathogenesis, respiratory and enteric syndromes in cattle remain persistent and complex challenges (6, 7). Conventional diagnostics, including microbial culture, microscopy, ELISA, and PCR, continue to serve as essential tools for pathogen detection. However, their reliance on prior assumptions about target organisms can limit their scope, often missing fastidious, novel, or low-abundance pathogens and providing only a partial view of the microbial communities (8, 9).

To address these limitations, culture-independent molecular approaches, particularly amplicon sequencing (metataxonomics) and shotgun metagenomics (SMGS), have emerged as powerful tools for characterising microbial diversity in clinical and environmental samples. For example, recent metagenomic studies have analysed faeces, wastewater and soil from dairy farm environments to detect antibiotic resistance genes and identified their associated bacterial hosts in livestock associated settings (10). Amplicon sequencing refers to the targeted sequencing of conserved genes with variable regions, such as 16S rRNA for bacteria, ITS-2 for helminths, and 18S rRNA for protozoa, to infer the composition and relative abundance of microbial communities. While informative, these methods are constrained by primer bias and limited taxonomic resolution. SMGS, by contrast, involves untargeted sequencing of all genetic material in a sample, enabling comprehensive detection of known and novel organisms, including viruses, bacteria, fungi, and parasites, without prior knowledge of the pathogens present (11–13).

This review explores how culture-independent sequencing technologies are reshaping our understanding of microbial diversity in bovine respiratory and enteric disease. We focus on the contributions of these approaches to pathogen detection and microbial community profiling, highlighting key findings across virology, bacteriology, and parasitology. Rather than resolving questions of disease causation, our emphasis is on how these genomic tools enhance the capacity to detect, characterise, and study microbial populations in cattle. We also discuss current limitations, particularly the lack of standardised bioinformatics pipelines and challenges of interpreting metagenomics findings in a clinical context and outline future directions to enhance the utility of these approaches in veterinary health and diagnostics.

## Viral diversity and detection in bovine respiratory and enteric disease

Viral pathogens are central to the pathogenesis of BRD and neonatal calf diarrhea. Current understanding of BRD describes pneumonia as a multifactorial condition, in which often viral infections compounded by environmental stressors predispose cattle to secondary bacterial colonisation, thereby exacerbating disease severity (14). The main respiratory viruses considered in differential diagnosis include DNA virus bovine herpesvirus-1 (BoHV-1) and RNA viruses such as bovine parainfluenza virus 3 (BPI-3), and bovine respiratory syncytial virus (BRSV) (15).

In enteric disease, bovine rotavirus A (BoRVA) has played a prominent role in neonatal calf diarrhea, while other viruses, including bovine adenovirus, bovine coronavirus (BCoV) and bovine viral diarrhea virus (BVDV) can affect both respiratory and gastrointestinal systems (16, 17). Detection of these viruses becomes the core goal of traditional diagnostic technologies (i.e., PCR, antigen detection, serology, virus isolation), which together have laid the foundation for infectious disease detection and surveillance in cattle.

### Viral detection overview

Accurate detection of viral agents is essential for disease control, outbreak investigation, and informed clinical treatment decisions in cattle (18). Validated detection methods including virus isolation, PCR, antigen ELISA, and serological assays remain central to veterinary virology. Each technique offers distinct advantages: serology is valuable for assessing past exposure and immune responses, while virus isolation enables confirmation of infectivity. PCR-based assays, including multiplex panels are widely used for the rapid and sensitive detection of common respiratory and enteric viruses (19–21).

To accommodate the complexity of polymicrobial infections and improve diagnostic throughput, many veterinary diagnostic laboratories have adopted multiplex PCR panels, capable simultaneously detecting multiple pathogens: viral, bacterial and/or protozoa associated with either enteric or respiratory disease (22, 23). Respiratory panels typically target BoHV-1, BVDV, BRSV, BPI-3, and BCoV, with some labs also including Influenza D Virus (IDV). Enteric panels commonly include BoRVA and BCoV in addition to common bacteria and protozoa (16, 20, 24).

While these panels are well-suited for high-throughput clinical settings and offer efficient detection of known pathogens, they are inherently limited by their predefined target list. This restricts their utility in identifying emerging, rare genetically divergent viruses, particularly in syndromes characterised by high microbial diversity and frequent coinfections, where known pathogens may only represent a portion of the infectious community. These limitations have prompted growing interest in untargeted sequencing approaches, which are now reshaping our understanding of viral diversity in cattle.

### Addressing the absence of universal genomic markers for viral detection

Unlike bacteria and fungi, viruses lack a universally conserved genomic region analogous to the 16S rRNA or ITS genes, which precludes the use of metataxonomic strategies for broad viral surveillance. To address this limitation, alternative methods have been developed to enable detection across multiple viral families. One such approach is pan-viral group PCR, which employs degenerate or consensus-degenerate hybrid oligonucleotide primers targeting conserved regions within essential viral genes, such as polymerases (25). These assays can sensitively detect diverse members within viral families or genera, including divergent or previously uncharacterised variants. For instance, Schlaberg et al. applied pan-viral group PCR targeting 19 viral families alongside RNA sequencing to uncover previously undetected pathogens in paediatric pneumonia cases lacking etiologic diagnosis, underscoring its utility for broad surveillance where standard diagnostics fail (26). In parallel, probe-based hybridisation capture methods such as ViroCap have been designed to enrich for viral nucleic acids from over 30 vertebrate-infecting viral families (27). These panels substantially increase viral read recovery by over 100-fold compared to SMGS and have demonstrated their value in both clinical and non-clinical contexts, including the detection of unexpected and novel viruses in wildlife mortality events. While these approaches offer increased speed and sensitivity relative to untargeted metagenomic sequencing, a key limitation is that they have been primarily optimised for human pathogens, potentially reducing their efficacy in veterinary or wildlife surveillance without deliberate adaptation or probe redesign (28).

### Metagenomics and the expanding virosphere

To capture the full spectrum of viral diversity beyond the constraints of targeted detection, untargeted sequencing approaches, particularly metagenomics and metatranscriptomics, have transformed viral discovery by enabling comprehensive assumption-free detection of nucleic acids directly from clinical and environmental samples (29, 30). These techniques allow for the simultaneous identification of known and novel viruses, expanding our understanding of viral diversity across ecosystems. The impact is evident in the rapid growth of recognised viral taxa: in 2015, the International Committee on Taxonomy of Viruses (ICTV) listed 3,704 species; by 2024, this number increased to 16,213 (31). While many of these viruses were identified from environmental or wildlife sources, an increasing number have been identified in livestock, raising questions about their possible relevance to animal health and disease ecology (32, 33). To aid interpretation, a general bioinformatics workflow and the potential biases associated with each analytical step are summarised in Supplementary Figure 1.

In cattle, metagenomic studies have uncovered a broad range of emerging viruses associated with both respiratory and enteric diseases. In BRD, previously understudied viruses such as IDV, Bovine rhinitis A virus (BRAV) and Bovine rhinitis B virus (BRBV) have been recurrently detected in animals presenting with respiratory pathology (34, 35). Another example of a potentially relevant BRD virus is Bovine nidovirus (BoNV), which was first assembled from lung samples during the investigation of a severe BRD outbreak in the United States in 2013. The virus was subsequently identified in nasal swabs from cattle in Australian feedlots, and more recently, it has also been detected in sheep presenting with respiratory disease (36–38). Although the pathogenic role of BoNV is uncertain, its emergence in across species and geographic regions underscores the value of broad-spectrum viral surveillance.

Similarly, metagenomic investigations into calf enteric disease have revealed a high prevalence of astroviruses, picornaviruses and caliciviruses, often detected in mixed infections. The identified viral families include *Caliciviridae* (e.g., bovine norovirus, nebovirus), *Picornaviridae* (e.g., bovine kobuvirus, enteroviruses E & F, hunniviruses, parechoviruses, bopiviruses, boosepiviruses); and *Astroviridae* (e.g., bovine astroviruses) (39, 40). Although the clinical relevance of many of these viruses remains unclear, their frequent detection warrants further investigation into their contribution to enteric disease. Advancing from detection to diagnosis will require studies assessing pathogenicity using either experimental or observational epidemiological designs to help clarify causal associations.

As sequencing technologies advance, enabling the detection of a broader range of viruses, previously hidden viral diversity is increasingly being revealed. The value of metagenomics for real-time untargeted surveillance is becoming more evident and increasingly recognised (41). A particularly impactful example is the recent detection of highly pathogenic avian influenza A (H5N1) in milk and nasal samples of cattle in North America. This event marked the first confirmed detection of influenza A in cattle and occurred on a farm located along a major migratory bird flyway, underscoring the potential for cross-species viral spillover and the need for sensitive, early-warning surveillance systems (42, 43). In parallel, farming practices such as high stocking densities, population mixing, and the selective pressures imposed by vaccination, may contribute to viral adaptation and evolution, further reinforcing the importance of ongoing metagenomic surveillance in livestock production systems (44, 45).

**Figure 1:**
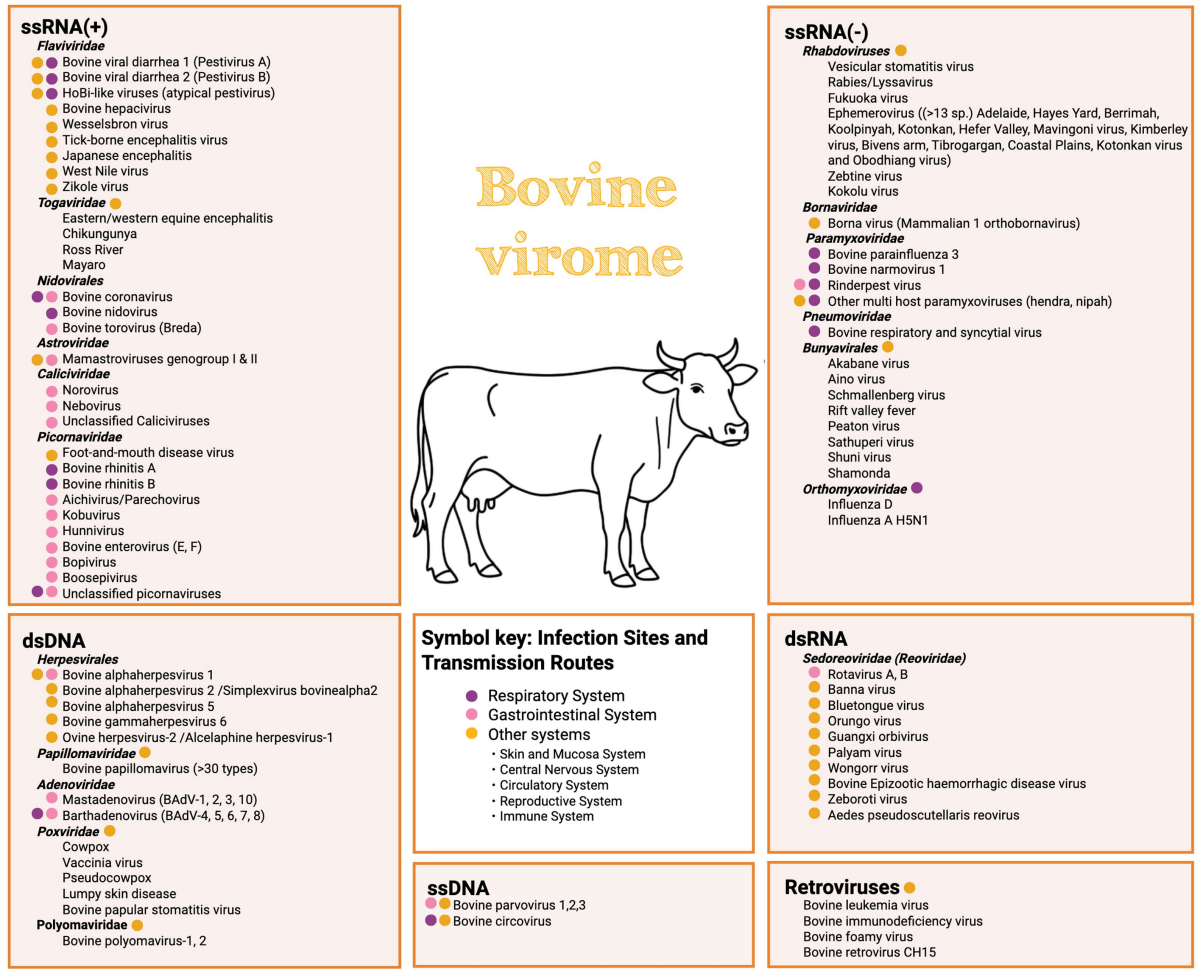
Summarises viruses detected in cattle, organised by genome type and taxonomic group. Colored dots indicate the primary infection sites or transmission routes. This overview highlights both viruses known to cause infection in cattle and those more recently detected in cattle, whose pathogenic potential or effects remain to be explored.

Although this review focuses on respiratory and enteric diseases, metagenomic investigations have also identified viruses associated with other organ systems, including neurotropic and vector-borne agents (46). These findings highlight the broader ecological scope of the bovine virome, with potential implications for immune function, disease susceptibility, and pathogen emergence. While not discussed in detail here, Figure 1 provides an overview of the viral species identified in cattle to date.

### Viruses: challenges and future directions

**Figure 2:**
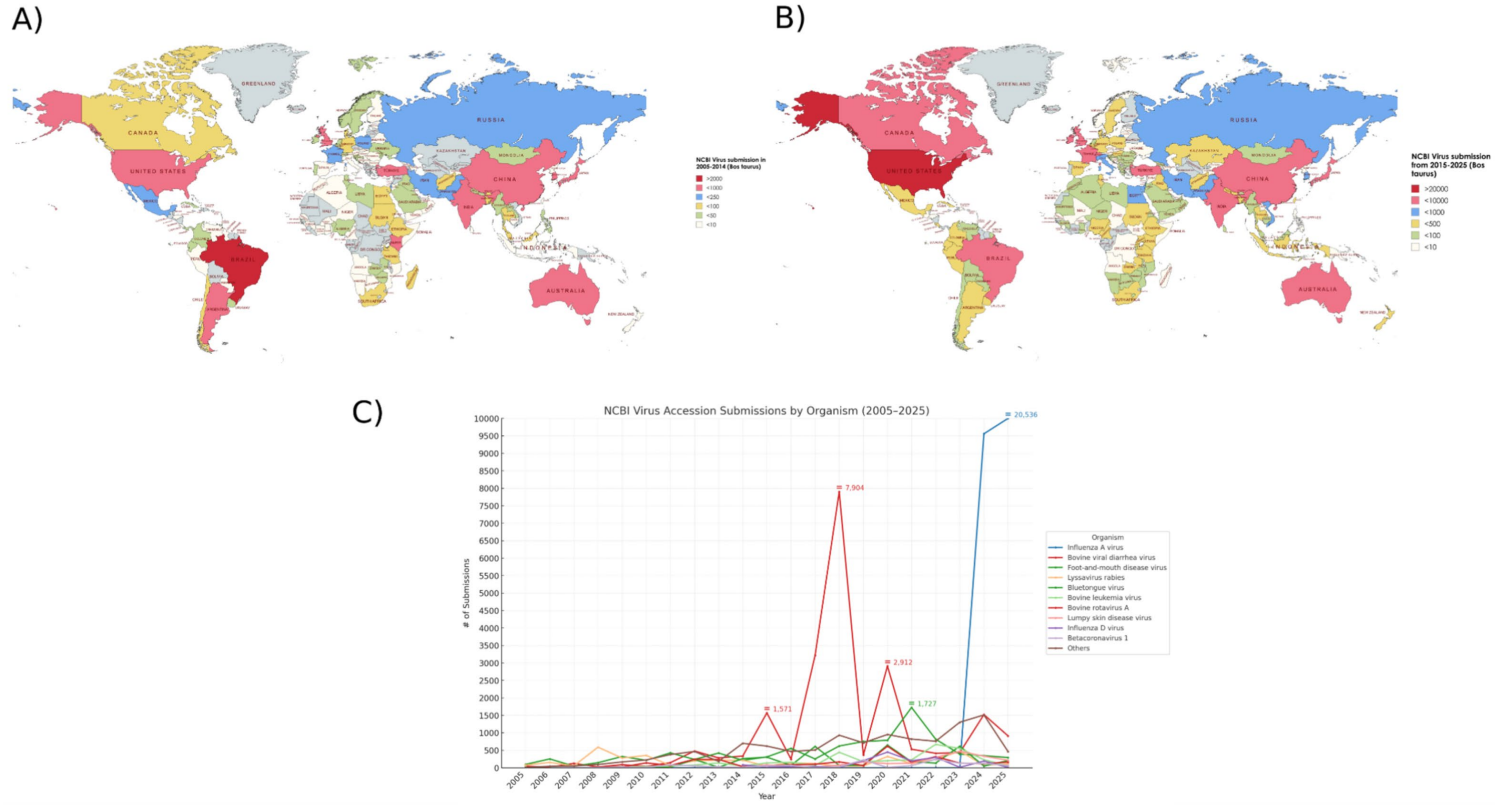
Global distribution and temporal trends of NCBI virus accession submissions associated with bovine. World maps show the number of submissions in 2005–2014 **(A)** and 2015–2025 **(B)**, with countries shaded according to submission counts (separate scales applied for each decade). The line graph **(C)** depicts annual submissions to NCBI by virus species from 2005 to 2025. The top ten most frequently submitted viruses are shown individually, with all others grouped as “Others.” The *y*-axis is scaled from 0 to 10,000 in 500 submission increments with years on *x*-axis. Influenza A virus exhibited a sharp rise in submissions, increasing from near zero to >20,000 between 2023 and 2025, largely reflecting recent outbreaks in the United States. Together, these data illustrate shifts in geographic and organism-level patterns of virus sequencing activity over the past two decades.

As climate change, land-use shifts, and increased wildlife-livestock interactions reshape viral ecosystems, untargeted sequencing will become increasingly central in early detection and surveillance efforts. Metagenomics has broadened our view of the bovine virome, revealing both established pathogens and novel viruses, some known only from sequence data. With recent changes in ICTV criteria allowing species designation without virus isolation, the pace of viral discovery has accelerated. Over the past two decades, cattle-associated virus submissions to NCBI have expanded markedly, reflecting both advances in sequencing technologies and shifts in global research priorities (Figure 2).

This rapid expansion underscores the critical importance of submitting high-quality, well annotated sequence data to public repositories. The utility of such databases depends not only on data volume but also on rigorous and ongoing curation, including accurate taxonomic assignment, standardised metadata and removal of erroneous or low-quality data entries. As large scale metagenomic outputs are only as informative as the contextual information that accompanies them, community-driven efforts to improve public database curation will be essential. Another major obstacle is distinguishing true host-associated viruses from environmental, dietary, or commensal contaminants, further highlighting the need for metadata-rich, curated reference sources.

To translate metagenomic discoveries into actionable insights, there is a growing need for integrative frameworks that combine high-throughput sequencing with downstream validation. Linking viral presence to disease phenotypes requires robust epidemiological data, as well as complementary tools such as *in situ* hybridisation, immunohistochemistry, and controlled infection studies (47). Moving forward, integrated approaches that bridge genomics, pathology, and epidemiology will be essential to realise the full potential of viral surveillance in cattle. These efforts must be supported by well curated public databases, as reliable classification, traceability and interpretation of viral sequences depend on consistent annotation and quality control. These efforts will not only improve disease diagnostics and outbreak preparedness but also enhance our broader understanding of viral ecology in livestock systems.

## The detection of helminths and protozoa

Parasitic infections are important contributors to cattle health and production challenges worldwide, particularly in enteric disease syndromes, muscle and hide damage and to a lesser extent, in respiratory conditions. Effective detection is essential for timely intervention, reducing production losses and mitigating broader health impacts.

Microscopy remains the foundation of traditional parasitological diagnostics, particularly for the identification of gastrointestinal parasites via faecal flotation, culture and sedimentation techniques (48). These methods are cost-effective and accessible but rely heavily on operator expertise and may lack sensitivity, particularly when parasitic stages (usually eggs) are shed intermittently or in low numbers. ELISA-based assays have also been employed to detect either parasite antigens or host antibodies. Antibody based ELISAs can support monitoring of prior exposure and associated productivity losses when performed regularly, usually monthly, intervals. However, their utility is limited by the fact that they do not indicate current infection and may lack specificity due to cross-reactivity among different parasite species (49, 50).

Molecular tools such as PCR and digital PCR (dPCR), offer enhanced sensitivity for detecting parasitic DNA in faecal or tissue samples. While PCR is often compromised by inhibitors present in faecal material, dPCR improves tolerance to these inhibitors better and allow for more accurate quantification. Nevertheless, both techniques are limited by their inability to distinguish viable from non-viable parasites, which constrains clinical interpretability (51–53).

Emerging genomic approaches, particularly metabarcoding and metagenomics, show promise for profiling parasite communities in cattle. However, their diagnostic integration into routine diagnostics remains limited, due to challenges in genome complexity, gaps in reference databases, and the inability to quantify infection burden (54).

### Helminths: complexity and diagnostic limitations

Helminth infections are widespread in cattle and can lead to a range of clinical outcomes, most commonly affecting the gastrointestinal tract. Gastrointestinal nematodes, including genera such as *Cooperia*, *Haemonchus, Ostertagia*, *Trichostrongylus* and *Oesophagostomum* are frequently implicated, often resulting in diarrhea, malabsorption, weight loss and poor growth rates. Other nematodes such as *Trichuris*, *Strongyloides* and *Toxocara*, may also be contribute to disease (55, 56). In the lungs, *Dictyocaulus* causes bronchitis, while zoonotic helminths such as *Echinococcus* spp. pose additional public health risks (57, 58).

Trematodes, such as *Fasciola hepatica* (liver fluke) and several species of *Paramphistomidae* (rumen fluke) are particularly problematic in grazing systems where they reduce milk production and contribute to chronic weight loss. Cestodes, such as *Moniezia* spp., transmitted via oribatid mites, primarily affect calves and are associated with reduced growth and development. Despite their clinical impact, helminth control is hindered by subclinical infections, inconsistent treatment and the emergence of anthelmintic resistance (59–61). A recent study demonstrated that early detection of resistance mutations is possible through deep amplicon sequencing and highlighted that increased use of benzimidazole in North American cattle could drive widespread resistance across multiple parasite species (62).

From a diagnostic standpoint, helminth infections present several complexities. Traditional methods such as faecal egg counts and larval cultures remain widely used, especially in mixed infections, but lack genus-level resolution and are subject to variability in egg shedding. Molecular approaches, particularly PCR and metabarcoding, offer higher sensitivity and taxonomic specificity, but share the limitations due to reliance on the same sample type (63).

Metabarcoding of the internal transcribed spacer 2 (ITS-2) region has proven effective for characterising gastrointestinal nematode communities, enabling genus- and species-level identification of key pathogens such as *Haemonchus*, *Trichostrongylus*, and *Cooperia* (11). While some mitochondrial markers, such as the 12S and 16S rRNA genes, and the cytochrome oxidase I (COI) gene, have been used to detect helminths in environmental samples, these techniques often lack specificity for distinguishing between pathogenic and non-pathogenic species (64). Moreover, the inability to differentiate viable from non-viable organisms, limits the clinical utility of these molecular methods, as previously discussed.

A recent study on strongylid nematodes illustrates both the promise and limitations of molecular diagnostic. High-throughput sequencing of the ITS-2 region of rDNA enabled nemabiome analysis, identifying 144 strongylid nematodes across 8 genera and 12 species. While this approach demonstrated the power of molecular tools in uncovering parasite diversity, it also underscored the ongoing challenges of applying these methods in routine clinical diagnostic settings (65). An overview of currently available genomic sequence data for nematodes and platyhelminths infecting cattle, including mitochondrial genomes, cytochrome oxidase genes, ITS-2 regions, and complete genomes, is provided in Table 1.

**Table 1:** Genomic data availability for bovine helminths.

Phylum	Class	Order	Family	Genus	Species	Mitochondrial genome	Cytochrome oxidase (partial or complete)	Complete genome	ITS2	18S rRNA	Common name	Primary host	Anatomic location	Pathogenicity
Nematoda	Chromadorea	Rhabditida	Ancylostomatidae	*Bunostomum*	*phlebotomum*	NC_012308.1	-	-	GQ859497.1	LC743846.1	Cattle hookworm	Cattle	Small intestine	Pathogenic
			Filariidae	*Parafilaria*	*bovicola*	-	MZ563429	-	MG983750.1	-	Summer bleeding	Cattle and Horse	Skin (causes focal cutaneous haemorrhages)	Pathogenic
				*Stephanofilaria*	*stilesi*	-	OP589133	-	-	OP596207.1	Skin worm	Cattle	Skin (causes dermatitis)	Pathogenic
					*sp*	-	MW143322	-	-	-	Skin worm	Cattle	Skin (causes dermatitis)	Pathogenic
			Metastrongylidae	*Dictyocaulus*	*viviparus*	-	-	GCA_964187775.1	-	-	Lungworm (verminous bronchitis)	Cattle, llamas, and alpacas	Lungs and bronchi	Pathogenic
					*filaria*	-	-	-	KF266761.1	KF266761.1	Lungworm (verminous bronchitis)	Small ruminants (goats, sheep, llamas, and alpacas)	Lungs and bronchi	Pathogenic
					*arnfieldi*	-	OP604478.1	-	OP605545.1	-	Lungworm (verminous bronchitis)	Equids only	Lungs and bronchi	Pathogenic
			Molineidae	*Nematodirus*	*oiratianus*	NC_024639	-	-	KR809574.1	-	Twisted wireworm	Cattle, sheep and goats	Small intestine	Pathogenic
					*spathiger*	NC_024638	-	-	KF305647.1	KT875352.1	Twisted wireworm	Sheep and goats (cattle is less common)	Small intestine	Pathogenic
					*helvetianus*	-	-	-	KC580753.1	-	Thin-necked intestinal worm	Sheep and goats (cattle are less common)	Small intestine	Pathogenic
			Onchocercidae	*Onchocerca*	*gibsoni*	MW579623.1	-	-	-	-	Filarial worm	Cattle	Subcutaneous tissues, ligaments	Pathogenic
					*gutturosa*	-	AJ271617	-	DQ317653.1	KP760156.1	Filarial worm	Cattle	Ligamentum nuchae	Pathogenic
					*armillata*	-	KX853322	-	OP605545.1	KX853334.1	Filarial worm	Cattle	Aortas	Pathogenic
					*lienalis*	-	KX853326	-	DQ317657.1	-	Filarial worm	Cattle	Gastrosplenic ligament	Pathogenic
					*ochengi*	-	-	GCA_000950515.2	-	-	Filarial worm	Cattle	Skin	Pathogenic
			Setariidae	*Setaria*	*digitata*	-	-	GCA_003640385.1	-	-	Filarial worm	Cattle (also infects sheep, goat and horses)	Peritoneal cavity and cerebrospinal cavity	Pathogenic
					*labiatopapillosa*	NC_044071	-	-	ON512459.1	KX853338.1	Filarial worm	Cattle, camels, horses, goats, and sheep (also wild mammals)	Abdominal cavity and blood stream	Non-pathogenic (sometimes cause mild fibrinous peritonitis)
					*marshalli*	-	LC719467	-	-	-	Filarial worm	Cattle	Abdominal cavity and blood stream	Non-pathogenic (sometimes cause mild fibrinous peritonitis)
			Strongylidae	*Chabertia*	*ovina*	NC_013831.1	-	-	JF680981.1	AJ920341.1	Large-mouthed bowel worm	Sheep and goats (sometimes cattle when co-grazed with sheep and goat)	Large intestine	Non-pathogenic
				*Oesophagostomum*	*columbianum*	NC_023933.1	-	-	JX188477.1	-	Nodular worm	Sheep and goats (cattle is less common)	Gastrointestinal	Pathogenic
					*asperum*	NC_023932.1	-	-	KM200806.1	AB971665.1	Nodular worm	Sheep and goats	Large intestine	Pathogenic
					*radiatum*	-	-	-	MG685662.1	-	Nodular worm	Cattle	Large intestinal (nodules- obstruction)	Pathogenic
					*dentatum*	-	-	GCA_000797555.1	-	-	Nodular worm	Pigs	Large intestine	Pathogenic
					*venulosum*	-	-	-	OR255926	-	Large bowel worm	Sheep and goats	Large intestine	Rarely pathogenic
			Strongyloididae	*Strongyloides*	*papillosus*	-	-	GCA_005656395.1	-	-	Threadworm	Sheep, goat and cattle	Gastrointestinal	Pathogenic
			Thelaziidae	*Thelazia*	*callipaeda*	-	-	GCA_900618365.1	-	-	Eyeworm	Domestic and wild carnivores	Eyes	Pathogenic
			Toxocaridae	*Toxocara*	*vitulorum*	NC_070176	-	-	KY442062.1	KJ398347.1	Roundworm	Cattle, and buffalo (rarely sheep and goats)	Small intestine	Pathogenic
			Trichostrongylidae	*Cooperia*	*oncophora*	-	-	GCA_036418165.1	-	-	Intestinal worm	Cattle	Small intestine	Pathogenic
					*punctata*	-	MW051257.1	-	MH267779.1	-	Intestinal worm	Cattle	Small intestine	Pathogenic
					*pectinata*	-	MH290297.1	-	MH267784.1	-	Intestinal worm	Cattle	Small intestine	Pathogenic
				*Haemonchus*	*contortus/placei*	-	-	GCA_000469685.2 GCA_900617895.1	-	-	Barber’s pole worm	Cattle, small ruminants and camelids	Abomasum	Pathogenic
				*Mecistocirrus*	*digitatus*	NC_013848.1	-	-	JQ863405.1	-	Large stomach worm	Cattle and Buffalo	Abomasum and intestines	Low pathogenic
				*Ostertagia*	*ostertagi*	-	-	GCA_964213895.1	-	-	Brown stomach worm	Cattle	Abomasum (gastroenteritis)	Pathogenic
					*lyrata*	-	MW051260.1	-	KX929995.1	-	Brown stomach worm	Cattle	Abomasum (gastroenteritis)	Pathogenic
					*leptospicularis*	DQ354359.1	AF044939.1	-	PP458236	KC998776	Stomach worm	Deer, cattle and other ruminants	Abomasum (gastroenteritis)	Pathogenic
				*Trichostrongylus*	*axei*	NC_013824.1			-	-	Stomach hairworm	Cattle, horses, swine	Stomach and intestines	Low pathogenic (not primary pathogen)
	Enoplea	Trichinellida	Capillariidae	*Aonchotheca*	*putorii*	NC_071371.1	-	-	-	LC052363.2	Gastrointestinal capillariid	Several mammals especially mustelids	Stomach and small intestine	Low pathogenic
			Trichuridae	*Trichuris*	*discolor*	NC_018596.1	-	-	OP824836.1	HF586910.1	Whipworm	Primarily cattle, also sheep and goats	Mucosa of cecum	Pathogenic
Platyhelminthes	Cestoda	Cyclophyllidea	Anoplocephalidae	*Moniezia*	*benedeni*	NC_036218	-	-	LC459963.1	GU817404.1	Tapeworm	Sheep, goats and cattle	Small intestine	Pathogenic (less evident)
					*expansa*	-	-	GCA_019097775.1	-	-	Tapeworm	Sheep, goat and cattle	Small intestine	Pathogenic (less evident)
				*Thysanosoma*	*actinoides*	-	-	-	-	-	Fringed tapeworm	Domestic and wild herbivores (cattle and sheep)	Bile ducts, pancreatic ducts and small intestine	Low pathogenic
			Taeniidae	*Taenia*	*saginata*	-	-	GCA_001693075.2	-	-	Beef tapeworm	Cattle	Gastrointestinal	Pathogenic
				*Echinococcus*	*granulosus*	-	-	GCA_000524195.1	-	-	Tapeworm	Dog (definitive host) but adult worms can be found in sheep, goats, swine, cattle, camels, horses, deer		Low pathogenic
	Trematoda	Plagiorchiida	Dicrocoeliidae	*Dicrocoelium*	*dendriticum*	-	-	GCA_944474145.2	-	-	Liver fluke	Cattle, alpacas and llamas	Abdominal cavity (may cause cirrhosis)	Low pathogenic
			Paramphistomidae	*Calicophoron*	*calicophorum*	-	JX678269.1	-	GU133057.1	L06566.1	Rumen fluke	Cattle	Rumen, reticulum and small intestine	Low pathogenic
				*Paramphistomum*	*ichikawai*	-	-	-	-	-	Rumen fluke	Cattle and sheep	Rumen, reticulum and small intestine	Low pathogenic
					*cervi*	NC_023095.1	-	-	KJ459938.1	KJ459938.1	Rumen fluke	Cattle and sheep	Rumen, reticulum and small intestine	Pathogenic
			Fasciolidae	*Fasciola*	*hepatica*	-	-	GCA_948099385.1	-	-	Liver fluke	Human, sheep, cattle, goats, buffalo, camelids and cervids	Liver, biliary ducts and gall bladder	Pathogenic
				*Fascioloides*	*magna*	NC_029481.1	-	-	EF051080.1	EF534989.1	Liver fluke	Deer, moose, elk, and cattle (cattle is dead end host)	Liver (hepatitis), bile ducts	Low pathogenic

This table compiles genomic data available for nematodes and platyhelminths infecting cattle. It summarises the presence of absence of relevant genetic markers mitochondrial genomes, cytochrome oxidase genes, complete genomes, ITS-2 regions, and 18S rRNA sequences, along with taxonomic classifications, common names, primary hosts, and the anatomical sites of infection.

### Protozoa: molecular diagnostics and genomic data gaps

Protozoan parasites are significant contributors to enteric diseases in cattle, particularly in young calves. In the gastrointestinal tract, genera such as *Eimeria, Cryptosporidium and Giardia* are frequently detected (66). *Cryptosporidium* spp. particularly *C. parvum*, are recognised as major cause of neonatal diarrhea in calves under two months of age and represent a significant zoonotic threat (67). Although other protozoa, including *Entamoeba*, are often identified in faecal samples, their pathogenic roles remain unclear (68). Compared to helminths, protozoan parasites represent a more tractable target for molecular assays, largely due to the availability of relatively well-established molecular markers. Beyond the gastrointestinal system, protozoan infections also affect the circulatory, reproductive and occasionally the central nervous systems. Tick-borne pathogens *Babesia* and *Theileria* cause systemic disease marked by haemolytic anaemia and fever (69, 70), while *Neospora caninum* and *Tritrichomonas foetus* are associated with reproductive loses including abortion and infertility (71, 72).

Molecular diagnostics, particularly PCR-based methods, have significantly improved the detection and differentiation of protozoan pathogens in cattle (73, 74). These tools offer high sensitivity and specificity, enabling detection at low pathogen loads and supporting genotyping and molecular subtyping, critical for phylogenetic analysis and epidemiological tracking (75). In *Entamoeba bovis*, molecular classification is primarily based on rRNA gene sequences, and recent studies have proposed further subdivision into ribosomal lineages (RL1,2 and RL4). Although the clinical relevance of these lineages is still being clarified, their distinction offers a valuable framework for species confirmation and evolutionary analysis (68). Genotyping of *Giardia* spp., particularly *Giardia duodenalis* into distinct assemblages provides insights into host specificity and zoonotic potential. The predominant genetic types in cattle are assemblage E, which is largely host-adapted to livestock, particularly ruminants, whereas assemblage A has a broader host range, infecting both livestock and humans (76–78). Similarly, *Cryptosporidium* species are classified into subtypes based on genetic markers such as the gp60 gene. Subtype families like *C. parvum* IIa are frequently detected in pre-weaned calves and are also among the most reported in human infections (78). Such genetic classifications not only support accurate diagnosis but also inform transmission dynamics within cattle populations and across species boundaries, aligning with One Health goals to understand the flow of pathogens between animals, humans, and shared environments.

Metabarcoding has emerged as a valuable tool for the simultaneous detection of multiple protozoan targets, particularly haemoparasites. Using primers that amplify near full-length 18S rRNA gene regions, this approach enables the identification of diverse taxa such as *Babesia*, *Hepatozoon*, *Neospora*, *Plasmodium*, *Theileria*, and *Toxoplasma* (79). Compared to traditional methods like blood smears, metabarcoding offers improved sensitivity and taxonomic resolution. However, the diagnostic performance of both molecular and microscopic techniques remains heavily influenced by the timing and quality of sample collection. In cases of transient parasitemia, where the parasite is intermittently present in the bloodstream, even the most sensitive assays may fail to detect the infection if parasitic stages are absent at the time of sampling.

Despite these advances, major challenges remain in the molecular detection and characterisation of protozoan parasites due to uneven genomic data availability. While extensive resources exist for zoonotic protozoa such as *Cryptosporidium* spp. (e.g., CryptoDB; https://cryptodb.org/cryptodb/app), many bovine-relevant pathogens still lack fully sequenced genomes. As summarised in Table 2, important genera such as *Eimeria* and *Sarcocystis* remain underrepresented in public genomic databases. Although treatment protocols may not always require species-level differentiation, the absence of complete genomic data hinders the development of species-specific molecular assays and limits the depth of molecular epidemiological studies. Expanding reference databases to include comprehensive genomic data for these underrepresented protozoan species is critical, not only for improving diagnostic accuracy and surveillance, but also for advancing our understanding of their biology, epidemiology, and potential zoonotic risks.

**Table 2:** Compiles of protist species known to infect cattle, highlighting the availability of their genomic data in publicly accessible databases.

Phylum	Class	Order	Family	Genus	Species	Cytochrome oxidase (partial)	Complete genome	ITS1	18S rRNA	Common name	Primary host	Anatomic location	Pathogenicity
Sarcomastigophora	Lobosa	Amoebida	Entamoebidae	*Entamoeba*	*histolytica*	-	GCA_000208925.2	-	-	Amoeba	Non-human primates, dogs, and cats (occasionally in cattle)	Large intestine and cecum	Pathogenic
					*bovis*	-	-	-	KT184336.1		Cattle, Horse, sheep, and goats	Rumen	Pathogenic
					*dispar*	-	GCA_000209125.2	-	-		Cattle and other ruminants	Rumen	Non-pathogenic
					*moshkovskii*	-	GCA_002914575.1	-	-		Horse (not mainly in cattle)	Rumen	Not mainly in cattle- not known if it’s a pathogen
					*ecuadoriensi*	-	-	-	-		Wild rodents and non-human primates	Free living	Not known if it’s a pathogen
					*invadens*	-	GCA_000330505.1	-	-		Reptiles	Gastrointestinal	Pathogenic
					*suis*	-	-	-	-		Pigs and Gorillas	Gastrointestinal	Non-pathogenic
Metamonada	Parabasalia	Trichomonadida	Trichomonadidae	*Tritrichomona*	*foetus*	-	GCA_905133005.1	-	-	Flagellate protozoa	Cat, and cattle	reproductive tract	Pathogenic
	Fornicata	Diplomonadida	Hexamitidae	*Giardia*	*intestinalis*	-	GCA_000002435.2	-	-	Flagellate protozoa	Dogs, cats, cattle, sheep, goats, horses, pigs	Small intestine	Pathogenic
Euglenozoa	Kinetoplastea	Trypanosomatida	Trypanosomatidae	*Trypanosoma*	*vivax*	-	-	-	-	Nagana	Cattle and wild mammals	Blood	Pathogenic
					*congolense*	-	-	-	-	Nagana	Cattle, sheep, pigs, goats, horses and camels, and dogs	Blood	Pathogenic
					*brucei*	-	-	-	-	African trypanosomiasis	Cattle, human	Blood and tissue cells	Pathogenic
Apicomplexa	Conoidasida	Eucoccidiorida	Cryptosporidiidae	*Cryptosporidium*	*parvum*	-	GCA_000165345.1	-	-	Cryptosporidiosis	Cattle (young lambs and goats)	Small intestine	Pathogenic
			Sarcocystidae	*Toxoplasma*	*gondii*	-	GCA_000006565.2	-	-	Toxoplasmosis	Cat and other warm-blooded animals (also infects sheep and cattle)	Reproductive system	Cattle can be infected but no abortion or perinatal mortality have been noticed
				*Neospora*	*caninum*	-	GCA_000208865.2	-	-	Neosporosis	Dog and cattle	Reproductive system (endogenous transplacental transmission in cattle)	Pathogenic
				*Besnoitia*	*besnoiti*	-	-	-	-	Besnoitiosis	Goat, horse, wild ruminants and cattle	Skin and reproductive system	Pathogenic
				*Sarcocystis*	*cruzi*	OR570880.1	-	EF622175.1	MH129611.1	Sarcocystosis	Cattle, sheep, pigs, horses, dogs, cats, and human	Gastrointestinal, eosinophilic myositis	Pathogenic
					*hominis*	-	-	-	-		Cattle, sheep, pigs, horses, dogs, cats, and human	Muscles (meat condemnation)	Pathogenic
					*heydorni*	-	-	-	-		Cattle and human	Muscle tissues	Pathogenic
					*hirsuta*	-	-	-	-		Cattle	Muscle tissues	Pathogenic
					*bovifelis*	-	-	-	-		Cattle	Muscle tissues	Pathogenic
					*bovini*	-	-	-	-		Cattle	Muscle tissues	Pathogenic
					*rommeli*	-	-	-	-		Cattle and water buffalo	Muscle tissues	Pathogenic
			Eimeriidae	*Eimeria*	*alabamensis*	OQ557081.1	-	-	-	Coccidiosis	Cattle	Gastrointestinal (neonatal diarrhea) and central nervous system (occurs in midwinters)	Moderately pathogenic
					*auburnensis*	OR42098	-	-	-				Moderately pathogenic
					*bovis*	KT184372.1	-	AB769731	KT184336.1				Highly pathogenic
					*zuernii*	OR039268	-	-	-				Highly pathogenic
					*brasiliensis*	-	-	-	-				Low pathogenicity
					*ellipsoidalis*	-	-	-	-				Moderately pathogenic
					*cylindrica*	-	-	-	-				Low pathogenicity
					*canadensis*	-	-	-	-				Low pathogenicity
					*pellita*	-	-	-	-				Low pathogenicity
					*subspherica*	-	-	-	-				Low pathogenicity
					*bukidnonensis*	-	-	-	-				Low pathogenicity
					*wyomingensis*	-	-	-	-			Small intestine (oocysts observed during a necropsy)	Non-pathogenic
	Aconoidasida	Piroplasmida	Babesiidae	*Babesia*	*bovis*	-	GCA_000165395.2	-	-	Bovine babesiosis	Cattle, water buffalo and deer	Blood	Mildly pathogenic
					*bigemina*	-	GCA_000981445.1	-	-		Cattle, water buffalo and deer	Blood	Mildly pathogenic
					*ovata*	-	GCA_002897235.1	-	-		Cattle	Blood	Mildly pathogenic
					*divergens*	-	GCA_001077455.2	-	-		Cattle, reindeer, and non-human primates	Blood	Mildly pathogenic
					*major*	-	-	EF422220.1	GU194290		Cattle	Blood	Not well known
					*occultans*	-	-	-	KJ000483.1		Cattle	Blood	Non-pathogenic
			Theileriidae	*Theileria*	*parva*	-	GCA_000165365.1	-	-	Theileriosis	Cattle	Lymph nodes	Pathogenic
					*orientalis*	-	GCA_003072545.4	-	-		Cattle	Lymph nodes (anaemia and mortality occasionally)	Non-pathogenic
					*annulata*	-	GCA_000003225.1	-	-		Cattle	Lymph nodes	Pathogenic
					*buffeli*	-	GCA_026724395.1	-	-		Cattle	Lymph nodes (anaemia and mortality occasionally)	Non-pathogenic
					*mutans*	-	-	-	-		Cattle	Lymph nodes	Pathogenic
					*velifera*	-	-	-	-		Cattle	Lymph nodes	Pathogenic

The table includes taxonomic classifications and the presence or absence of genetic sequences such as whole genomes, mitochondrial genomes, cytochrome c oxidase genes (partial or complete), or internal transcribed spacer (ITS) regions. The majority of the species listed belong to the phylum Apicomplexa, with representatives from the families *Theileriidae*, *Eimeriidae*, *Sarcocystidae*, *Babesiidae*, and *Cryptosporidiidae*. Additionally, two species from the phylum Metamonada (*Giardia* and *Tritrichomonas*) and several species from the Amoebozoa genus *Entamoeba* are included. Notably, no full genomes exist for any *Eimeria* or *Sarcocystis* species infecting cattle. For species lacking a complete genome, the table documents whether partial genetic data are available, such as ITS regions, cytochrome c oxidase genes, or mitochondrial genomes. This compilation underscores significant gaps in genomic resources for several pathogenic protists, emphasising the need for further sequencing efforts to improve molecular detection, characterisation, and epidemiological studies using modern metabarcoding and metagenomics approaches.

### Diagnostic challenges and future directions for molecular detection of enteric and respiratory parasites in cattle

Despite advances in molecular parasitology, significant challenges hinder the routine use of genomic tools in cattle. Helminth diagnostics are limited by large, polymorphic genomes and a lack of high-quality reference data, which complicates species identification and resistance monitoring. While protozoa are more amenable to molecular assays, uneven genomic representation (i.e., *Eimeria* and *Sarcocystis*) restricts assay development. Metabarcoding and metagenomics offer broader surveillance potential but remain underutilised due to high costs, bioinformatics demands, and limited standardisation. Additionally, validated molecular markers for anthelmintic resistance are scarce beyond the benzimidazole class. Expanding reference genome databases, improving affordability, and developing field-adaptable protocols will be essential to integrate these tools into routine diagnostics and advance sustainable parasite control strategies in cattle.

## Bacterial diversity in bovine respiratory and enteric disease: insights from metagenomics and amplicon sequencing

Bacterial pathogens remain central to research and diagnostics in bovine respiratory and enteric disease. Traditional diagnostics, including microbial culture, microscopy, and PCR, have played a foundational role in detecting well-known pathogens such as *Mannheimia haemolytica, Pasteurella multocida, Histophilus somni,* and *Mycoplasma bovis* in cases of BRD*, Escherichia coli, Clostridium perfringens* and *Salmonella* spp. in case of enteric diseases (80). However, these methods are inherently selective. Culture conditions often fail to support fastidious or low-abundance organisms, and PCR panels are limited by predefined targets, potentially missing unexpected taxa. While culture remains indispensable for phenotypic characterisation and antimicrobial susceptibility testing, it inherently restricts the scope of detection to organisms that grow under laboratory conditions. As a result, the microbial composition captured by these methods may not fully reflect the diversity or relative abundance of pathogens present in clinical samples, potentially skewing our understanding of complex disease etiologies. In contrast, culture-independent approaches provide a complementary perspective, enabling untargeted detection of fastidious, low-abundance, or previously unrecognised bacteria directly from clinical samples.

### Metagenomics and amplicon sequencing: complementary, culture-independent tools

Culture-independent sequencing has opened new avenues for investigating the bovine microbiome. Three approaches have gained traction:

Amplicon sequencing, typically based on 16S rRNA gene sequencing, allows broad taxonomic profiling of bacterial communities directly from clinical samples, bypassing the need for culture. By targeting conserved regions of the 16S rRNA gene, this method can detect both dominant and low-abundance taxa, offering insight into overall microbial communities and shifts associated with disease. Recent advances in sequencing technologies, including both short- and long-read platforms, have improved its taxonomic resolution. However, 16S rRNA sequencing often lacks the precision to distinguish closely related species and cannot provide functional data, such as virulence or resistance gene content. Additionally, it depends on existing reference databases, which limit its ability to detect novel or poorly characterised organisms (81, 82). Despite these constraints, 16S rRNA gene sequencing remains a valuable tool for initial detection and characterisation of the microbial populations and can serve as a foundation for deeper analysis via metagenomics.
SMGS involves sequencing all DNA present in a clinical sample, enabling comprehensive, untargeted analysis of microbial communities. Unlike targeted approaches, it captures the full genomic content, including both abundant and rare organisms allowing for high-resolution taxonomic identification alongside functional profiling. This includes detection of genes associated with antimicrobial resistance (ARGs), virulence factors (VFs), and other traits relevant to host-pathogen interactions (83, 84). Despite these advantages, SMGS remains constrained by high costs, and significant laboratory and bioinformatic expertise requirements (85).
Metatranscriptomics, which sequences RNA instead of DNA, adds an additional layer by identifying genes that are actively expressed. This provides insight into the metabolic activity and functional state of microbial populations at the time of sampling (86). When combined with metagenomics, these approaches may offer a powerful means to characterise complex microbial ecosystems in cattle. However, this approach comes with its own limitations. Sample collection can be destructive and requires sufficient material for sequencing. Moreover, metatranscriptomics may fail to capture the complete transcriptional landscape due to microbial complexity, wide variation in transcript abundance, RNA instability, and technology-specific constraints, and it does not inherently preserve genomic context or guarantee species-level taxonomic resolution (87).

### Investigating underrecognised bacterial pathogens in BRD and enteric disease

Metagenomic and metatranscriptomic approaches allow population-level investigations into microbial community dynamics, helping to distinguish incidental findings from disease-associated shifts. While earlier reports of atypical bacterial species in BRD often originated from isolated case studies relying on culture, recent metagenomic and metataxonomic investigations are beginning to clarify their broader prevalence (88). For example, several reports have documented a diverse range of less commonly considered bacteria in BRD-affected animals, including *Gallibacterium* spp., *Chlamydia pecorum*, *Alysiella* spp., *Helcococcus ovis*, *Acinetobacter* spp. and *Pseudomonas fluorescens* among others (89–93). Such data points to the potential involvement of underappreciated taxa in BRD pathogenesis. However, determining the population-level impact of these rarely reported or atypical bacterial taxa in BRD will require larger-scale, hypothesis-driven investigations.

In this context, genome-resolved metagenomics has emerged as a critical tool for uncovering underrecognised bacterial diversity in bovine respiratory and enteric systems. Metagenome-assembled genomes (MAGs), reconstructed from shotgun sequencing data using short- and increasingly hybrid short- and long-read approaches, allow partial or near-complete genomes to be recovered directly from complex microbial communities without prior cultivation. Recent large-scale studies in cattle have demonstrated that a substantial proportion of enteric and respiratory bacteria remain genomically uncharacterised at the species level, with more than half of recovered MAGs lacking assignment to known species (94). This highlights a major knowledge gap in bovine microbiology and suggests that many bacteria potentially relevant to health, disease susceptibility, or microbial community stability remain invisible to both culture-based diagnostics and marker-gene surveys. Importantly, MAGs provide access not only to taxonomic novelty but also to functional potential, including metabolic pathways, virulence-associated traits, and antimicrobial resistance genes, offering a mechanistic framework for prioritising candidate pathogens or dysbiosis-associated taxa for further investigation (95). As such, genome-resolved metagenomics represents a key bridge between microbial discovery and hypothesis-driven studies aimed at clarifying causal roles in bovine respiratory and enteric disease.

Furthermore, untargeted tools offer an opportunity to differentiate closely related bacterial species and strains, such as *Mycoplasma* spp. While *Mycoplasma bovis* is frequently targeted in diagnostics, other species like *M. dispar* and *M. bovirhinis* may also play roles in respiratory disease but are often overlooked. Similarly, taxa such as *Moraxella bovoculi*, *M. oculi*, and *M. bovrei*, or *Mannheimia* var*igena* and *M. pernigra,* may differ in prevalence and pathogenicity, yet are rarely distinguished by routine diagnostics. Metagenomic methods could clarify their individual contributions and improve disease attribution.

Enteric disease studies have similarly expanded the list of candidate pathogens. Untargeted sequencing of diarrheic calves has revealed *Campylobacter* and various *Helicobacter* spp. and other bacteria, suggesting possible roles in subclinical or underdiagnosed gastrointestinal disorders. These findings challenge the prevailing reliance on *E. coli, Clostridium perfringens*, and *Salmonella* spp. as the primary bacterial culprits in calf diarrhea.

The detection of such underrecognised organisms raises important questions. Are these taxa genuinely rare, or are they simply missed by traditional diagnostics? This should be answered in the near future with the broader application of untargeted sequencing methods to uncover the full range of bacteria contributing to disease.

## Future challenges and opportunities

Despite the growing power of sequencing, culture-dependent tools continue to offer critical complementary advantages. Matrix-Assisted Laser Desorption/Ionization - Time-of-Flight Mass Spectrometry (MALDI-TOF MS) enables rapid identification of cultured isolates with minimal bioinformatics needs, though its utility is restricted by reference database coverage (96). Culturomics extends this by using diverse growth conditions to isolate fastidious or previously unculturable bacteria, followed by high-throughput identification (97). The whole genome sequencing (WGS) of individual bacteria, while reliant on prior isolation, provides high-resolution insight into strain-level variation, virulence, and antimicrobial resistance, particularly useful in outbreak investigations (98). For instance, the WGS of *Mycoplasma bovis* isolates from cattle has revealed extensive genomic diversity, resistance determinants, and virulence-associated genes, enabling detailed epidemiological tracing during outbreaks (99).

By expanding our ability to detect and characterise diverse bacterial communities, culture-independent approaches will provide a deeper understanding of the microbial dynamics underlying bovine respiratory and enteric diseases. Unlike culture, which often favours fast-growing organisms and misses fastidious or unculturable taxa, sequencing methods capture the microbial range in a semi-quantitative manner that more closely reflects the community structure present in the host.

Moving forward, metagenomic data should guide the development of targeted diagnostic tools, such as expanded PCR panels, for taxa that are confirmed or strongly suspected to play a significant role in disease, enabling their integration into routine surveillance and improving the diagnostic coverage of bovine respiratory and enteric syndromes. For instance, some studies have incorporated bovine kobuvirus, and norovirus into multiplex PCR panels for the diagnosis of neonatal calf diarrhea, based on their frequent detection in metagenomic surveys of diarrheic calves (100, 101). These additions complement traditional PCR panels that typically target pathogens such as bovine coronavirus, bovine rotavirus, *Escherichia coli* K99, *Salmonella* spp., and *Cryptosporidium*, thereby enhancing the breadth and sensitivity of pathogen detection in clinical settings. In parallel, robust epidemiological studies and experimental infection models will be essential to determine whether these organisms act as incidental colonisers or true causative agents of disease. Such integrative strategies will help translate microbial discovery into actionable insights for disease management and control. Methodological heterogeneity also remains a major challenge for the comparability of metagenomic studies. Differences in sample collection and handling, including sampling site, depth of collection, nucleic acid extraction protocols, library preparation, sequencing depth and downstream bioinformatics processing such as host / non microbial reads removal strategies, parameter settings and taxonomic databases can substantially influence microbial profile and may compromise the reproducibility of the results.

Metagenomic next-generation sequencing (mNGS) has transformed our ability to investigate the microbial ecosystems underlying major cattle syndromes, including calf diarrhea and BRD. By enabling broad-spectrum detection of bacteria, viruses, and parasites, mNGS provides insights that extend beyond the scope of traditional diagnostics, particularly in multifactorial diseases where diverse agents contribute to clinical outcomes.

Yet, the value of mNGS lies not in detection alone, but in meaningful interpretation. Microbial nucleic acids do not equate to disease causation; clinical expression reflects a complex interplay of pathogen load, virulence factors, host immunity, and environment. Moving forward, integrative frameworks that connect genomic data with pathology, epidemiology, and clinical observation will be critical. At present, the main barrier is the need of mature analytic pipelines particularly those that tailor outputs to clinicians and providing decision frameworks to distinguish clinical relevant findings. When combined with harmonised laboratory protocols and cross-disciplinary input, these developments will ensure that genomic discoveries are translated into actionable insights and become more clinical useful.

Equally important, the bioinformatics backbone of mNGS demands standardisation and rigor. From read processing to taxonomic classification and genome assembly, each step introduces potential biases that must be controlled to generate robust conclusions. For example, genome-resolved approaches such as metagenome-assembled genomes (MAGs) have expanded the analytical scope of mNGS by enabling the reconstruction of draft genomes from complex communities, providing access to uncultured taxa and their metabolic potential beyond what marker-gene or culture-based methods allow. However, assembly artefacts, incomplete reconstructions and challenges in accurate binning remain significant limitations, highlighting the need for improved algorithms, deeper sequencing and hybrid long and short read strategies (102). In parallel advances in artificial intelligence may offer opportunities to streamline and strengthen these pipelines and algorithms, including those supporting MAG reconstruction by enhancing pattern recognition and automating interpretation and quality control, thereby accelerating the incorporation of mNGS into routine diagnostics.

Ultimately, mNGS should not be viewed as a replacement for conventional tools but as a powerful complement that expands the diagnostic horizon. Its true potential lies in bridging discovery and application: uncovering the hidden diversity of the bovine microbiome, informing the design of targeted assays, and strengthening surveillance systems. By embedding mNGS within integrative diagnostic and epidemiological frameworks, we can move toward a more precise, predictive, and preventive approach to cattle health.

## Conclusion

Recent advances in sequencing technologies have reshaped our understanding bovine respiratory and enteric diseases by uncovering extensive microbial diversity that underlies these complex syndromes. Metagenomics and amplicon sequencing approaches have revealed both known and previously uncharacterised taxa, many of which were undetectable by conventional diagnostics. These tools have highlighted the polymicrobial nature of disease, expanded the repertoire of candidate pathogens, and underscored the importance of considering microbial ecology in disease pathogenesis. At the same time, culture-based methods, targeted molecular diagnostics, and isolate-level whole genome sequencing continue to provide essential insights into antimicrobial resistance, strain variation, and transmission dynamics.

Looking ahead, the effective translation of metagenomics discoveries into clinical practice will depend on the development of robust analytical pipelines, harmonised laboratory workflows and integrative frameworks that links genomic data with pathology, epidemiology and clinical context.

As these components mature, mNGS has the potential not only to expand diagnostic coverage but also to inform more precise, predictive and preventive strategies for disease management in cattle. Through the combined use of genomic and conventional tools, a more comprehensive and actionable understanding of bovine health can be achieved while metagenomic approaches continue to mature.

## Glossary

ARGs: Antimicrobial resistance genes
BRD: Bovine respiratory disease
BoHV-1: Bovine herpesvirus-1
BVDV: Bovine viral diarrhea virus
BPI-3: Bovine parainfluenza virus 3
BRSV: Bovine respiratory syncytial virus
BoRVA: Bovine rotavirus A
BCoV: Bovine coronavirus
BRAV: Bovine rhinitis A virus
BRBV: Bovine rhinitis B virus
BoNV: Bovine nidovirus
COI: Cytochrome oxidase I gene
dPCR: Digital PCR
IDV: Influenza D Virus
ICTV: International Committee on Taxonomy of Viruses
ITS-2: Internal transcribed spacer 2
MALDI-TOF MS: Matrix-Assisted Laser Desorption/Ionization - Time-of-Flight Mass Spectrometry
mNGS: Metagenomic next-generation sequencing
MAGs: Metagenome-assembled genomes
SMGS: Shotgun metagenomics
VFs: Virulence factors
WGS: Whole genome sequencing
