# Supplementary material for: Next-generation detection in bovine respiratory and enteric diseases: metagenomic and amplicon sequencing insights into microbial diversity
Source: Front Vet Sci. 2026 Apr 7;13:1788101. doi: 10.3389/fvets.2026.1788101 (PMC13097308; doi:10.3389/fvets.2026.1788101)
Supplement: Supplementary file 1 [file Image_1.pdf]

Next-Generation Detection in Bovine Respiratory and Enteric Diseases: Metagenomic and Amplicon Sequencing Insights into Microbial Diversity

Authors: Zain Ul Abedien, Ian J. Lean, Steven P. Djordjevic, Paul M. Hick, Mark E. Westman, Janina Mckay-Demeler, John Webster, Barbara Brito

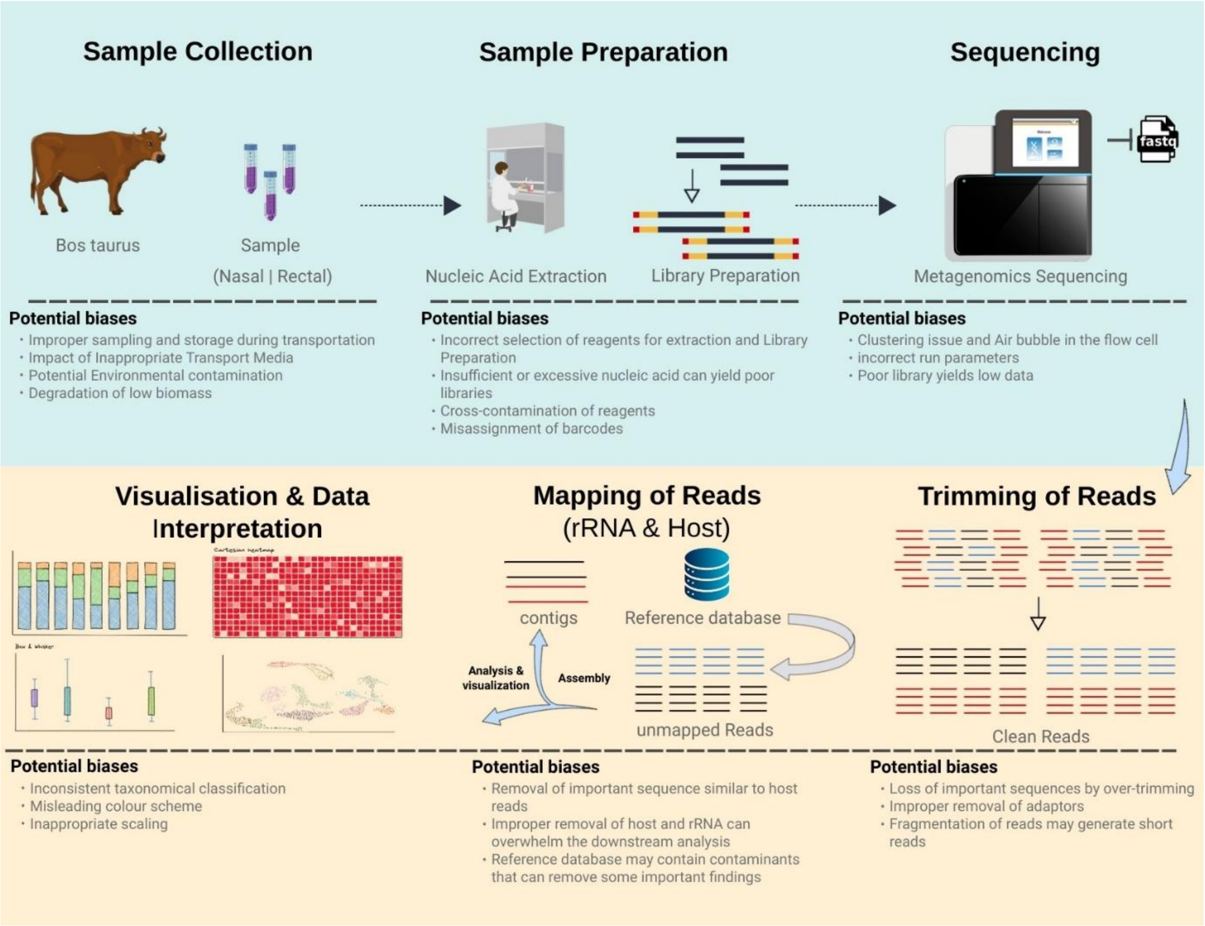

Supplementary figure 1. This schematic illustrates the key steps involved in metagenomic sequencing of bovine nasal and rectal samples, highlighting potential sources of bias at each stage of the workflow, from sample collection to bioinformatic interpretation.

Sample Collection: Proper handling of nasal and rectal samples is critical to avoid degradation or contamination. Potential biases include improper sampling or storage during transportation, inappropriate transport media, introduction of environmental contaminants, and low microbial biomass that can affect downstream nucleic acid recovery and sequencing depth.

Sample Preparation: During nucleic acid extraction and library preparation, errors such as incorrect reagent choice, insufficient or excessive input material, barcode

misassignment, and cross-contamination of reagents can compromise library quality and representation. These factors may skew taxonomic or functional profiles.

Sequencing: During the sequencing process, technical issues such as flow cell clustering errors, air bubbles, suboptimal run parameters, and low-yield libraries may introduce noise or reduce sequencing coverage, limiting detection of low-abundance taxa.

Trimming of Reads: Post-sequencing read processing involves quality filtering, adaptor trimming, and removal of short or low-complexity reads. Over-trimming may lead to loss of biologically relevant sequences, while under-trimming may retain artifacts. Improper fragmentation of reads can also affect assembly and alignment accuracy.

Mapping of Reads: Cleaned reads are mapped against reference databases to remove host and rRNA sequences and to identify microbial taxa. However, overly aggressive removal can inadvertently discard pathogen-derived sequences that are similar to host DNA. Furthermore, incomplete or contaminated reference databases may misclassify or overlook novel organisms.

Data analysis, visualization & interpretation: Final data analysis and visualization can be influenced by scaling artifacts, inconsistent taxonomic classification, and inappropriate choice of abundance estimation. These biases may distort biological interpretation and hinder comparability across studies.

Collectively, this figure underscores the importance of rigorous quality control and standardization at each step of the mNGS workflow to ensure accurate microbial profiling in bovine disease studies.
